# Prediction of activity and specificity of CRISPR-Cpf1 using convolutional deep learning neural networks

**DOI:** 10.1186/s12859-019-2939-6

**Published:** 2019-06-13

**Authors:** Jiesi Luo, Wei Chen, Li Xue, Bin Tang

**Affiliations:** 1 Department of Pharmacology, Key Laboratory for Aging and Regenerative Medicine, School of Pharmacy, Southwest Medical University, Luzhou, Sichuan China; 2 Center for Bioinformatics and Systems Biology and Department of Radiology, Wake Forest School of Medicine, Winston-Salem, NC 27157 USA; 3 School of Public Health, Southwest Medical University, Luzhou, Sichuan China; 4 Basic Medical College of Southwest Medical University, Luzhou, Sichuan China

**Keywords:** CRISPR, Guide RNAs design, Deep learning

## Abstract

**Background:**

CRISPR-Cpf1 has recently been reported as another RNA-guided endonuclease of class 2 CRISPR-Cas system, which expands the molecular biology toolkit for genome editing. However, most of the online tools and applications to date have been developed primarily for the Cas9. There are a limited number of tools available for the Cpf1.

**Results:**

We present DeepCpf1, a deep convolution neural networks (CNN) approach to predict Cpf1 guide RNAs on-target activity and off-target effects using their matched and mismatched DNA sequences. Trained on published data sets, DeepCpf1 is superior to other machine learning algorithms and reliably predicts the most efficient and less off-target effects guide RNAs for a given gene. Combined with a permutation importance analysis, the key features of guide RNA sequences are identified, which determine the activity and specificity of genome editing.

**Conclusions:**

DeepCpf1 can significantly improve the accuracy of Cpf1-based genome editing and facilitates the generation of optimized guide RNAs libraries.

**Electronic supplementary material:**

The online version of this article (10.1186/s12859-019-2939-6) contains supplementary material, which is available to authorized users.

## Background

The clustered regularly interspaced short palindromic repeats (CRISPR)-CRISPR-associated proteins (Cas), originally derived from bacterial adaptive immune systems [1–3], has become the center of attention since the invention of CRISPR-Cas9-based genome engineering technology [4–6]. After that, a dazzling line of CRISPR-Cas9 applications quickly emerged: genome-scale knockout/activation/repression screening [7–9], epigenome editing [10], base editing [11, 12], live-cell RNA imaging [13], gene drive [14] and many other applications. Despite the huge success of the CRISPR-Cas9 tool in genome editing, the demand for more precise and robust CRISPR-based tools is still growing [15]. Several recent efforts have focused on exploring the power of alternative CRISPR-Cas systems [16–18].

CRISPR-Cas systems can be classified into two distinct classes and further subdivided into at least six types [19–21]. The class 1 CRISPR-Cas systems (including type I, III, and IV) are found in diverse bacterial and archaeal phyla, comprising about 90% of the CRISPR-Cas loci. The remaining 10% of the CRISPR-Cas loci belong to class 2 CRISPR-Cas systems (including type II, V, and VI), which are found in diverse bacterial phyla but virtually absent in archaea [17]. An additional difference between class 1 and class 2 CRISPR-cas systems is the organization of effector module. Class 1 systems form multi-protein effector complexes to achieve RNA-guided nucleic acid targeting and degradation, whereas class 2 systems rely on a single-protein effector [19]. The relatively simple architecture of effector complexes has made the class 2 systems an attractive choice for use in the new generation of genome-editing tools.

Recently, a Cas protein named Cpf1, which belongs to the class 2 type V CRISPR-Cas system, has been repurposed for genome editing applications [22, 23]. Cpf1 has differences from Cas9 in several aspects. First, Cpf1 is a single crRNA nuclease that does not require a tracrRNA. Second, Cpf1 recognizes thymidine-rich PAM sequence at the 5’end of the protospacer region. Third, Cpf1 cleaves target DNA distal to the PAM site and produces cohesive (not blunt) ends with 4- or 5-nt overhangs [24–26]. Fourth, Cpf1 has a conserved RuvC nuclease domain, but lacks the HNH domain. Fifth, Cpf1 processes its own crRNAs [27]. These distinguishing features of Cpf1 make it a useful tool for enriching the CRISPR-based genome editing toolkit, broadening the spectrum of targetable genomic sites.

It’s time consuming and laborious to test all guide RNAs before staring a gene-editing experiment. In silico guide RNAs design has accordingly become a key issue for successful genome-editing. A number of online tools and applications have been developed for the design of guide RNAs. There are also several excellent reviews and articles comprehensively summarizing and benchmarking these tools [28–31]. Despite considerable efforts to date, predicting the activity and specificity of guide RNAs is still a challenge. In addition, most of tools and methods are developed for Cas9. The number of tools and methods for Cpf1 is relatively limited. Therefore, there is an urgent need to develop new computational tools for Cpf1.

In this work, we propose a deep learning approach to design Cpf1 guide RNAs. Our approach of using two convolutional neural networks classifiers stems from classification strategies used in image classification [32], where a first classifier predicts on-target activity using the matched DNA sequences and a second classifier predicts off-target effects using the mismatched DNA sequences. Each classifier is composed of a combination of “one-hot” feature representations. To capture the important characteristic of functional guide RNAs, we present the permutation importance analysis on the neurons extracted by the convolution and pooling processes, and map top neurons to original input matrix. We find that the seed region of guide RNA sequences determines target activity and specificity.

## Results

### DeepCpf1 architecture

**Fig. 1:**
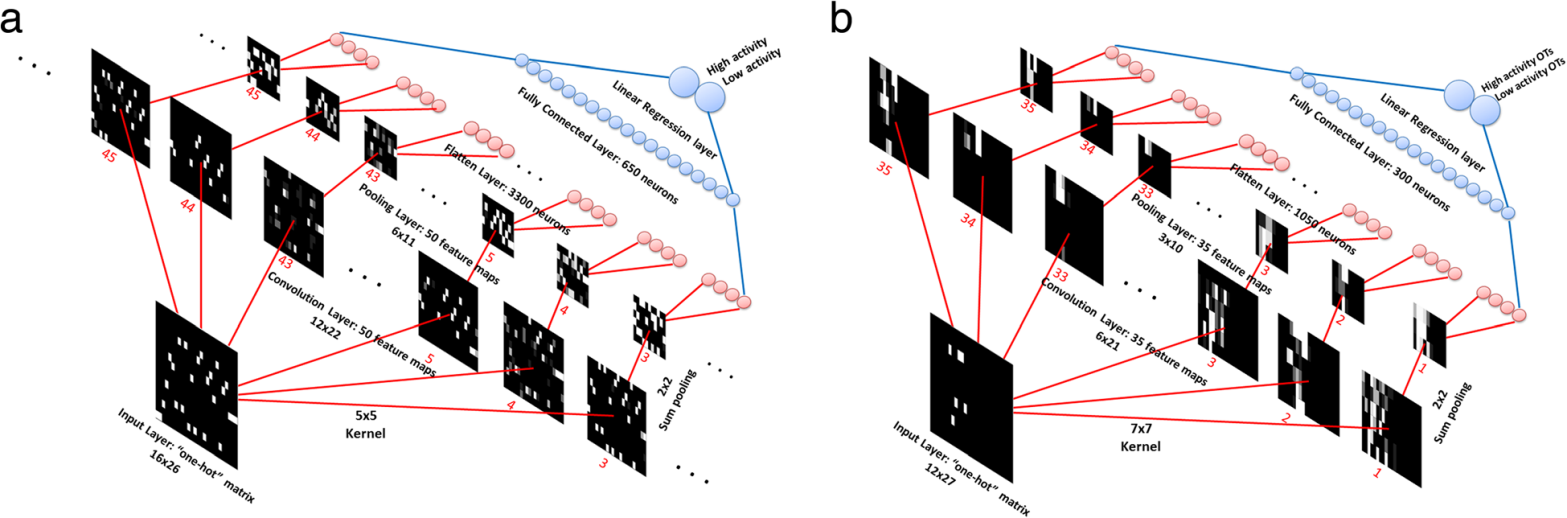
Inside the DeepCpf1 architecture. Data flow is from the lower left to upper right. The DNA sequence is translated into a “one-hot” matrix as original input (white indicates 1 and black indicates 0). The convolution and pooling operations are applied to the input and produces the output of each layer as feature maps. The feature maps are visualized as gray scale images by the image function in R. **a** on-target activity prediction. **b** off-target specificity prediction

The DeepCpf1 was built and trained using the MXNet framework in the *R* environment on a standard PC. The training architecture of the DeepCpf1 is given in the Fig. 1. The input layer for on-target activity prediction is a “one-hot” matrix with a size of 16 × 26 (Fig. 1a). The first convolutional layer performs 50 convolutions with 5 × 5 filter on the input layer, producing 50 feature maps of size 12 × 22. The second pooling layer performs 2 × 2 spatial pooling for each feature map using the sum value, and produces 50 new feature maps with a size of 6 × 11. The flatten layer reshapes the output of pooling layer into a 1-dimensional vector comprising 3300 neurons. A fully connected layer receives the output of the flatten layer and contains 650 neurons. Finally, the output of the fully connected layer is fed to a linear regression layer that assigns a score for the on-target activity. The off-target effects classifier has a CNN architecture similar to the on-target activity classifier (Fig. 1b). The 35 filters of size 7 × 7 are applied to the input in the first convolutional layer, followed by a pooling layer taking the sum value of 2 × 2 regions. The flatten layer and fully connected layer are composed of 1050 and 300 neurons, respectively.

### DeepCpf1 predicts Cpf1 activities using matched target sequences

Many interrelated architectural factors determine the performance of the convolutional network model, including the number of layers, feature map dimensions, number of parameters, etc. Therefore, the model architecture must be carefully designed and sized to make it appropriately for our purpose. Here, we focused on assessing the independent contributions of three important factors: kernel sizes, the numbers of feature maps per layer, and the numbers of layers. For other factors, we chose rectified linear units (ReLU) to follow each convolutional layer and performed sum-pooling after each convolution and rectification (Additional file 1: Figure S1a). We first provided an overview of the model’s performance at different kernel sizes (Additional file 1: Figure S1b). To assess the effect of variation in kernel sizes, we held fixed the numbers of layers and feature maps. The 5 × 5 kernel size showed the best performance as compared to the other sizes. Next, we tested the effect of varying the number of feature maps while holding fixed the numbers of layers and kernel sizes (Additional file 1: Figure S1c). The best performance was achieved when using 50 feature maps. We finally compared the average performance of one stage (comprising one convolutional layer and one pooling layer) and two stages (comprising two convolutional layers and two pooling layers) (Additional file 1: Figure S2), but did not find an improvement in their performance as the number of layers increases (Additional file 1: Figure S1d).

**Fig. 2:**
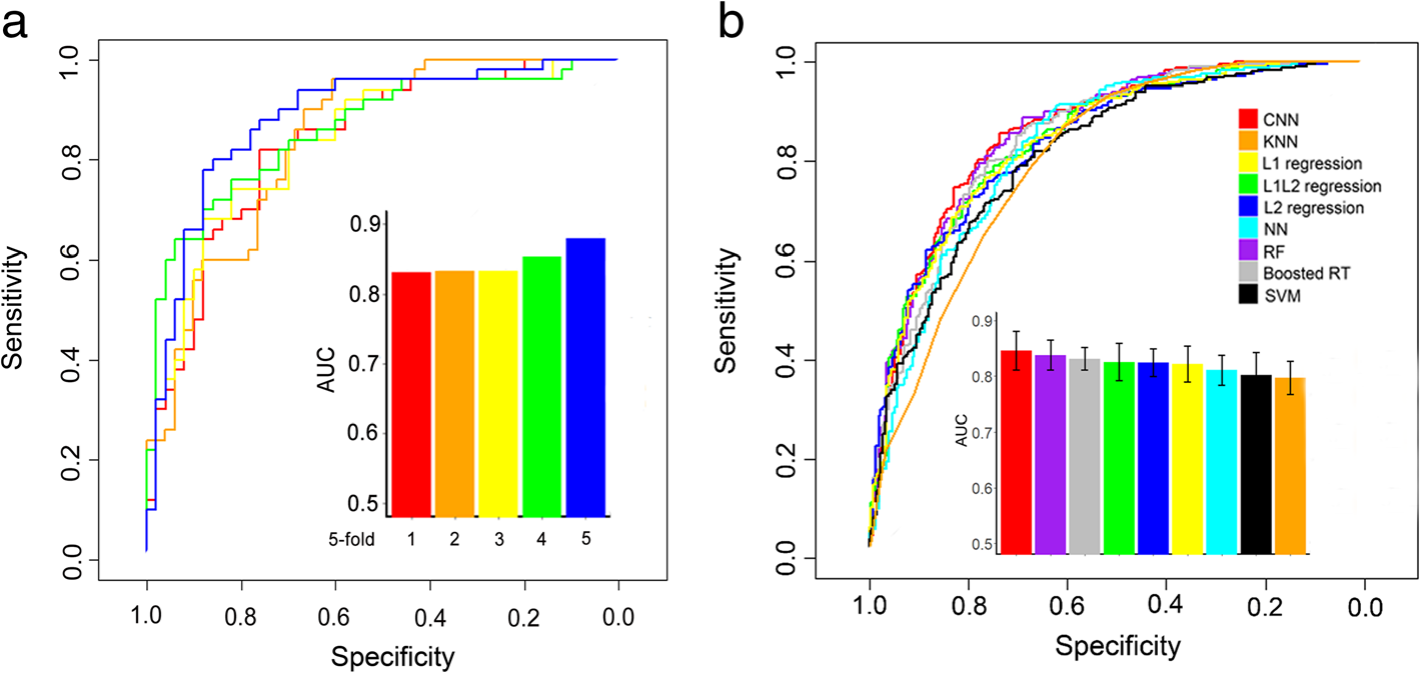
Prediction of Cpf1 guide RNAs on-target activities using deep convolutional neural networks. **a** ROC curves showing the predictive power of the DeepCpf1. Fivefold external cross-validation strategy was employed. **b** ROC curves and AUC values comparing the performance of the CNN and other machine learning methods

The implemented model architecture is shown in Fig. 1a in detail. For the total data set (size = 1251), forty-five convolutional network models were trained to separate potent and weak guide RNAs, which were pre-classified based upon different top- and bottom-efficacy cutoffs, respectively. As seen in Additional file 1: Figure S3, by excluding guide RNAs with modest activities, the functional guide RNAs can be more readily predicted. Thus, the CNN model was used to make a binary classification of the top 20% most effective guide RNAs versus the bottom 80% effective guide RNAs. In order to avoid over-fitting, the classifier was validated using a 5-fold external cross-validation procedure. Briefly, the entire dataset was randomly divided into five equal parts. Each of the five parts was left out in turn to form an external set for validating the model developed on the remaining four parts. This procedure was repeated five times in which every sequence in the dataset was predicted. We used standard values for the base rate of learning (0.005), momentum (0.9) and batch size of 40 examples to train the CNN classifier. The predictive performance has been estimated by the area under the curve (AUC) of the receiver operating characteristic (Fig. 2a). Our classifier achieved high AUCs of 0.846 ± 0.03 (mean ± s.d.) on five external test sets, indicating the robustness and reproducibility of the convolutional network model.

We compared the performance of several different “one-hot” encoding modes. We implemented CNN-order1 and CNN-order3 using the same training architecture, but the input matrix size was 4 × 27 and 64 × 25 instead of 16 × 26. It is worth noting that the 5 × 5 kernel size exceeds the dimension of 4 × 27 input matrix. Therefore, we performed 4 × 1 convolution on the input matrix, and followed by the 1 × 2 pooling in the CNN-order1 classifier. The CNN-order2 had better performance (0.846 and 0.77 mean AUC and F1, respectively) than did the CNN-order1 (0.78 and 0.67) and CNN-order3 (0.79 and 0.70) (Additional file 1: Figure S4). In addition, we found that the dimension of input matrix was correlated to the running time (Additional file 1: Figure S5). We further randomly rearranged the order of adjacent pairwise nucleotides 100 times for each input matrix to estimate the effect of row order. New inputs also enabled high-performance prediction with 0.83 AUC and 0.75 F1, indicating that the row order had little effect on performance (Additional file 1: Figure S4). Finally, we explored whether the performance of our model would be affected by neighbor sequences around guide RNAs binding sites. New input sequences are 40 bp in length, including the 23-bp guide sequences, 4-bp PAM sequences as well as seven nucleotides upstream and six nucleotides downstream of the guide RNAs binding sites. Although the CNN-order2-40 bp outperformed the CNN-order1-27 bp and CNN-order3-27 bp, it was not superior to the CNN-order2-27 bp (Additional file 1: Figure S4).

The 5-fold cross-validation was conducted to compare the performance of the CNN method with other machine learning methods, including Neural Network (NN), k-nearest neighbor (KNN), Support Vector Machine (SVM), Random Forest (RF), L1-regularized linear regression (L1 regression), L2-regularized linear regression (L2 regression), L1 L2-regularized linear regression (L1 L2 regression) and Gradient-boosted regression tree (Boosted RT). For SVM, we considered the radial basis function (RBF) as the kernel function, and two parameters, the regularization parameter C and the kernel width parameter γ were optimized by using a grid search approach. It could identify good parameters based on exponentially growing sequences of (C, γ) (C = 2^− 2^, 2^− 1^, …, 2^9^ and γ = 2^− 6^, 2^− 5^, …, 2^5^). The KNN algorithm needed to set the number of neighbors (K) in the set {3, 5, 7, 9, 11, 13, 15, 17, 19, 21 and 23} and the K with the highest prediction performance was kept. The standard feed-forward neural network was used, with a sigmoid transfer function and an optimal number of hidden layer neuron. The back-propagation algorithm was applied in training the NN, with random initial weights. The learning rate was set to 0.0001 and the weight decay to − 0.001. For RF, the two parameters, *ntree* (the number of trees to grow) and *mtry* (the number of variables randomly selected as candidates at each node), were optimized using a grid search approach; the value of *ntree* was from 500 to 3000 with a step length of 500, and the value of *mtry* was from 2 to 40 with a step length of 2. For linear regression (L1, L2 and L1 L2), the regularization parameter range was set to search over 100 points in log space, with a minimum of 10^− 4^ and a maximum of 10^3^. The Gradient-boosted regression trees used the default setting. All machine learning algorithms were implemented by the scikit-learn package in python and the predicted values in the Additional file 1: Figure S6 were obtained using the average values of 5-fold cross-validation from the results of parameter optimization process.

**Fig. 3:**
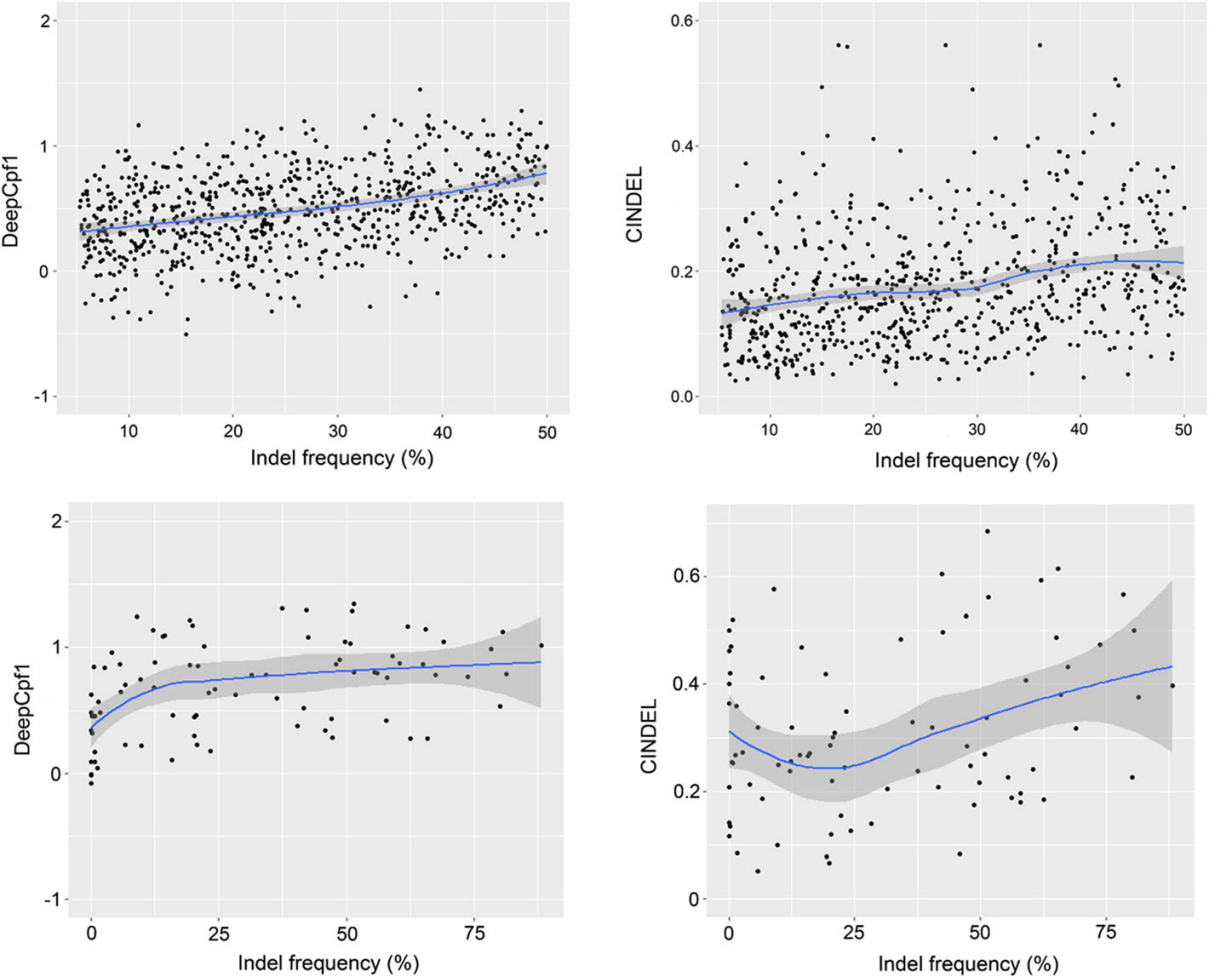
The scatter plots showing the correlation of the predicted scores and the indel frequencies of guide RNAs for test data set and independent data set

Using the same “order 2” features, the performance of different methods is shown in Fig. 2b. CNN outperformed the other eight methods and the AUC scores of 0.846, 0.838, 0.832, 0.825, 0.824, 0.821, 0.811, 0.802 and 0.797 were achieved for CNN, RF, Boosted RT, L1 L2 regression, L2 regression, L1 regression, NN, SVM and KNN, respectively. Next, we tested whether the DeepCpf1 model was informative to predict the indel frequencies of test data (Fig. 3). The activities of the 751 guide RNAs were predicted with DeepCpf1 and correlated to their indel frequencies. Furthermore, the performance of another design tool, CINDEL [33] was also evaluated using the same test set. The predicted efficiency scores of DeepCpf1 showed stronger positive correlation with indel frequencies compared with CINDEL. The spearman correlation coefficients (*R*) were 0.38 for the DeepCpf1 and 0.27 for CINDEL, respectively. The general applicability of both methods were further evaluated using the independent AsCpf1-induced indel frequency data obtained from 84 guide RNAs. The DeepCpf1 and CINDEL predicted indel frequencies for guide RNAs with *R* = 0.33 and 0.27, respectively, in Fig. 3.

**Fig. 4:**
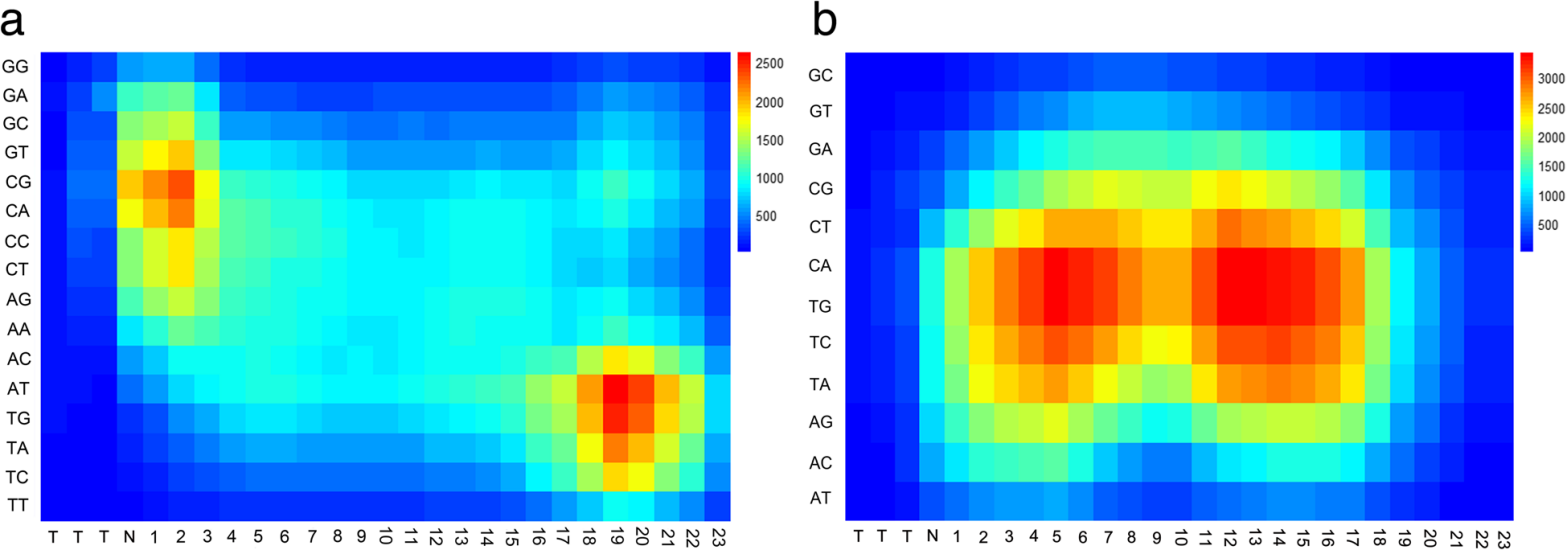
The top features to the CNN classifier for predicting Cpf1 activities at **a** matched target sequences and **b** mismatched target sequences

To further characterize the features of highly active guide RNAs, we performed the feature analysis on the 3300 neurons of flatten layer. We determined the feature importance by estimating the average decrease in node impurity after permuting each predictor variable. We analyzed the top features and mapped them from the flatten layer to the input matrix (Fig. 4a). We observed that most of top features were generated by convolving the upper left region of input matrix, where the thymine pairs were significantly depleted at the positions adjacent to the PAM. This result provides strong evidence that the seed sequence of guide RNAs affects CRISPR/Cpf1 efficacy through nucleotide compositions. A recent study has shown that Cpf1 pre-orders the seed sequence of the crRNA to facilitate target binding [34]; however, thymine in the seed sequence might destabilize interactions between the Cpf1 protein and crRNA [33]. In addition, we observed that the PAM-distal region of guide RNAs was also crucial for prediction, suggesting that the guide RNAs expression level was also an important factor when choosing highly active guide RNAs. Finally, we used the kpLogo web tool [35] to visualize the nucleotide differences between the top and bottom 20% guide RNAs (Additional file 1: Figure S7). The result is consistent with our feature analysis.

### DeepCpf1 predicts Cpf1 activities using mismatched target sequences

The proposed network architecture for specificity prediction is illustrated in Fig. 1b. The network comprises of only one convolutional layer, one pooling layer and one fully-connected layer with a small number of neurons. Here, we focused on disentangling and assessing the independent effects of two variables: the numbers of feature maps and kernel sizes (Additional file 1: Figure S8). 35 filters of size 7 × 7 were chosen and applied to the input in the first convolutional layer, followed by a ReLU and a sum pooling layer taking the sum value of 2 × 2 regions.

**Fig. 5:**
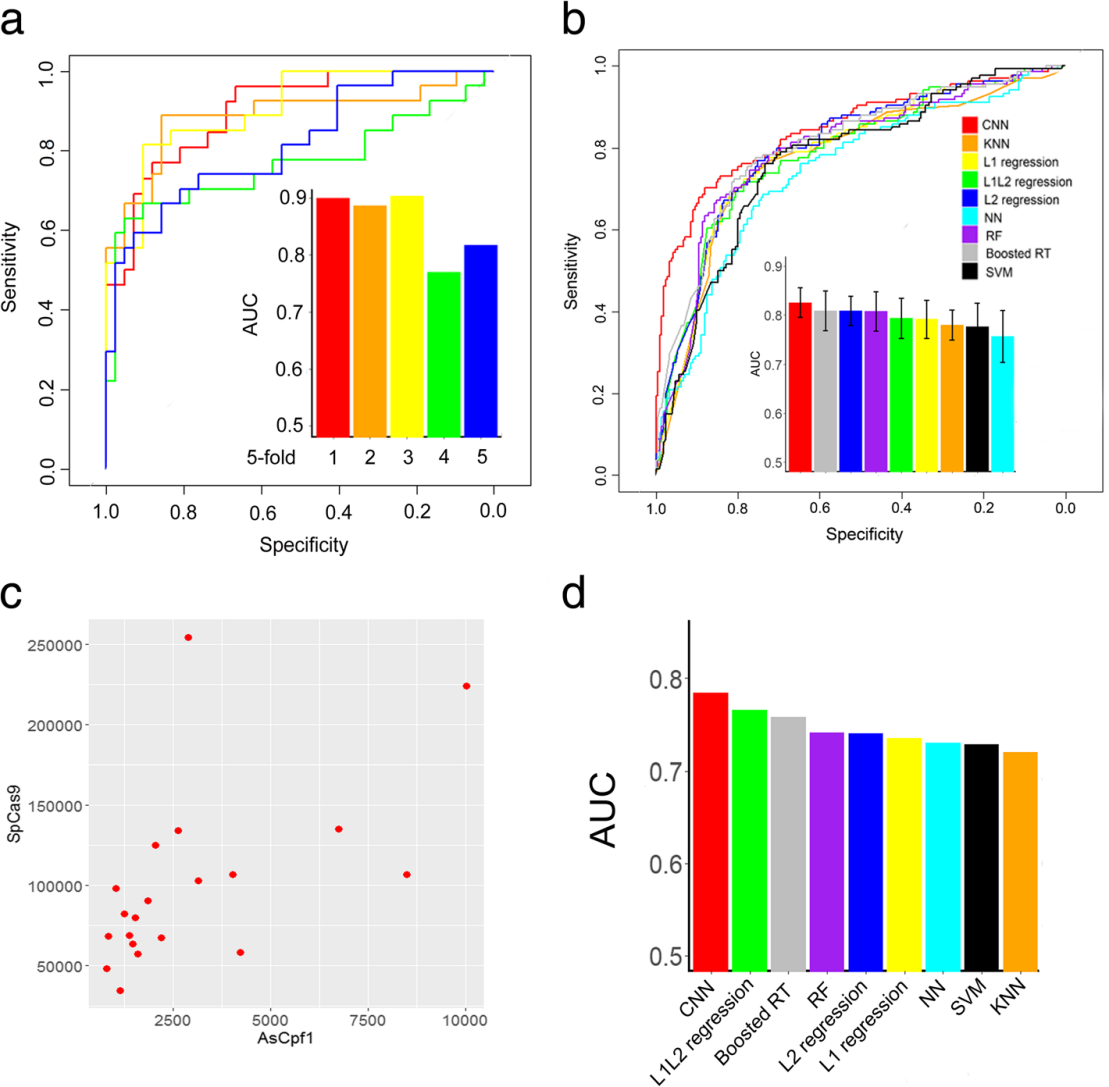
Prediction of Cpf1 guide RNAs off-target specificities using deep convolutional neural networks. **a** ROC curves used to assess the performance of DeepCpf1 with fivefold external cross-validation. **b** ROC curves and AUC values comparing the performance of the CNN and other machine learning methods. **c** Comparison of the genome-wide specificities of AsCpf1 and SpCas9 nucleases. All possible sites with up to seven mismatches to 20 guide RNAs were identified by Cas-OFFinder tool. **d** The performance of various machine learning methods on independently generated Digenome-seq and GUIDE-seq data sets

We evaluated the performance of the CNN classifier by using 5-fold external cross-validation. Strikingly, our classifier was able to distinguish highly active off-target sites from control off-target sites with high accuracy (Fig. 5a, mean AUC, 0.826). We next compared CNN to several additional machine learning approaches, including Boosted RT, L2 regression, RF, L1 L2 regression, L1 regression, KNN, SVM and NN. When trained on the same data with the same features, CNN outperformed the other methods and their AUC scores reached 0.826, 0.809, 0.808, 0.807, 0.794, 0.792, 0.780, 0.776 and 0.757, respectively. (Fig. 5b and Additional file 1: Figure S9).

The genome-wide specificities of Cpf1 nucleases are distinct from those of Cas9 nucleases, owing to their different modes of target recognition and PAM requirements. To roughly compare their specificities, we used Cas-OFFinder [36] to identify all possible sites with seven or fewer mismatches to the 20 endogenous human gene target sites that shared common protospacer sequences for both AsCpf1 and SpCas9 nucleases. We observed that Cpf1 nucleases contained much fewer off-target sites in comparison to Cas9 nucleases (Fig. 5c), which is in line with previous studies that Cpf1 nucleases were highly specific in human cells [22, 37]. We further evaluated whether the CNN classifier could predict the off-target sites obtained with Digenome-seq and GUIDE-seq, two experimental approaches for detection of crRNA target sites [22, 37]. Kleinstiver et al. carried out GUIDE-seq experiments with two Cpf1 nucleases in U2OS human cells using 19 crRNAs [37]. Kim et al. used a total of eight crRNAs and performed Digenome-seq experiments to identify all genome-wide Cpf1 off-target sites in vitro [22]. There were two crRNAs (*DNMT1* site 3 and site 4) overlap between two studies. For the *DNMT1* site 4, both methods showed no detectable off-target sites. Although the off-target sites of *DNMT1* site 3 identified by GUIDE-seq were also detected by Digenome-seq, some were unique to Digenome-seq and showed some differences in two methods [22, 37]. We carefully examined all off-target sites and removed the duplicate sites as well as the sites that cannot be found in the human genome. We finally found 26 and 50 off-targets sites obtained using GUIDE-seq and Digenome-seq methods, respectively. In addition, a total of 858 false off-target sites that differed from the crRNAs by up to six nucleotides were found using Cas-OFFinder [36]. We collected these true and false off-target sites as an independent data set to evaluate different machine learning algorithms. The CNN classifier obtained the highest AUC value of 0.784 for the independent data set (Fig. 5d).

Similarly, we evaluated the feature importance reported by the permutation importance analysis on the flatten layer. We found that the top features were mainly extracted from the bottom of the input matrix, where the C or G base of guide RNA sequences mismatched with target DNA sequences (Fig. 4b). Kim et al. segmented the protospacer sequences into three regions: seed (positions 5–10, where position 1 is located to the left in the input matrix), trunk (positions 11–22), and promiscuous (positions 23–27), according to the effect of base-pairing mismatches at each position [33]. They observed that the mismatches in the seed and promiscuous regions strongly and slightly decreased indel frequencies, respectively; whereas the trunk region mismatches reduced indel frequencies to an intermediate level. Our feature analysis results support this conclusion that the seed and part of promiscuous regions are most important for the target specificity.

## Discussion

To validate DeepCpf1 in the design of guide RNAs libraries, we took the protein-coding genomic sequence of TADA1, an essential gene for cell viability in cancer and pluripotent stem cells [38], from the UCSC Genome Browser and used DeepCpf1 to screen for both CRISPR activity and specificity. First, targetable sites for Cpf1 were identified by searching for genomic sequence matching TTTN-N23 motif. Next, the targetable sites that contained polyT and extreme GC content (< 30% or > 70%) were removed and the on-target activity scores of the remaining targetable sites were predicted by the DeepCpf1. The top 10% targetable sites ranked by the activity scores were further retained for predicting their off-target specificity scores. The scores were calculated based on the number of predicted high activity off-target sites. Finally, the optimized libraries were designed to maximize the activity scores and minimize off-target effects (Additional file 1: Figure S10).

Recently, Kim et al. used the deep learning to improve the prediction of CRISPR-Cpf1 guide RNA activity, and showed better performance than the previous methods from the DNA sequences [39]. Deep learning is a form of machine learning that uses a synthetic neural network architecture composed of interconnected nodes in multiple layers that can be trained on input data to perform a task. The high performance of deep learning is based on its ability to automatically extract sequence signatures, capture activity motifs and integrate the sequence context. Our work further extends the use of deep learning to the prediction of Cpf1 off-target sites. Different from previous off-target sites prediction methods, we used the one-hot encoding to translate the off-target sites in each position as a twelve-dimensional binary vector, in which each element represented the type of mismatch. The one-hot encoding is very suitable for the numerical representations of off-target sites, which can truly reflect the information about number, position and type of the mismatch. In addition, the CNNs can allow computers to process spatial representations of one-hot matrices efficiently and holistically, without relying on laborious feature crafting and extraction. The two deep learning models developed for the Cpf1 guide RNAs activity and specificity prediction are combined to create optimized guide RNA libraries that maximize on-target activity and minimize off-target effects to enable more effective and efficient genetic screens and genome engineering.

## Conclusion

We present DeepCpf1, a deep learning framework for predicting the activity and specificity of CRISPR-Cpf1 that explicitly captures nucleotide dependencies between guide RNA positions. We use two convolutional neural network based models, inspired from deep learning work in image recognition applications and validate them by comparing their predictions with outcomes of high-throughput profiling experiments. In addition, we use the permutation importance analysis to extract important combinatorial relationships between sequence positions and sequence compositions from the trained models. Our findings not only validate previous observations but also provide new insights for intrinsic on or off-target mechanisms. We expect that this tool will assist in reducing the numbers of Cpf1 guide RNAs that need to be experimentally validated to identify potent and specific guide sequences for a given target gene.

## Methods

### Materials

In a recent study, Kim and his colleagues established a lentiviral library of Cpf1 guide RNA-target sequence pairs [33]. They used this library to determine PAM sequences and evaluate the activity of Cpf1 with various guide RNA sequences at matched and mismatched target sequences. In our study, 1251 matched and 344 mismatched target sequences cleaved by Acidaminococcus sp. BV3L6 (AsCpf1) were collected from this published data set to develop our deep learning models. For the on-target activity prediction, the 1251 matched target sequences were first sorted by indel frequencies in descending order. Next, the data were split into training (40%, size = 500) and test (60%, size = 751) data. The training data, representing the most effective guides (top 20% in ranking) and the least potent guides (bottom 20%), were used for model architecture design and external cross-validation, and the test data were used to test the model’s ability to predict the indel frequencies of the remaining guide RNAs. An additional independent test data set of indel frequencies at 84 endogenous target sites was used to assess the generalization power of deep learning model. For the off-target effects prediction, we assigned the top 20% of mismatched sequences the class ‘High activity off-target sites’; the remaining 80% were assigned ‘Low activity off-target sites’. We assumed that the mismatched sequences with top 20% on-target cleavage efficiencies were more likely to induce off-target mutagenesis in vivo compared to the remaining 80%.

### Encoding the DNA sequences by the “one-hot” strategy

Here, the “one-hot” encoding refers to translating a nucleotide sequence into a two-dimensional numerical matrix, where each number can take on the value 0 or 1. For example, we used a window of 2 nucleotides and slid it through a 27-bp target sequence with a step of 1 nucleotide. The 27-bp sequence thus got converted to a 16 × 26 matrix; the row representing the position information of each nucleotide and the column representing all adjacent pairwise nucleotides, such as AA/AT/AC/AG/etc. These are “order 2” features [40]. Similarly, for other order features, we slid the sequence using different window sizes. How to accurately describe the mismatch information of each off-target sequence is a key issue for off-target effects prediction. Previous prediction algorithms can roughly be categorized into two classes: some simply use sequence alignment with mismatch counts to exhaustively search for off-target sites [36, 41, 42], while others use a specificity score calculation on the basis of a matrix of mismatch weights, obtained empirically, that reflects the importance of each position on cleavage efficiency [40, 43, 44]. Taking inspiration from the “one-hot” encoding, we translated each mismatch sequence into a 12 × 27 matrix, which truly reflected the information about number, position and type of the mismatch. In the matrix, the row represents the mismatch position and the column represents mismatch type, such as AT/AC/AG/etc.

### Convolutional neural network

Convolutional neural networks (CNN) were originally inspired by Hubel and Wiesel’s seminal work on the cat’s visual cortex [45]. LeCun introduced the computational architecture of CNN, which has been applied with great success to the detection, segmentation and recognition of objects and regions in images [46]. The typical architecture of CNN is composed of a series of stages. Each stage is structured as three types of layers: a convolutional layer, a non-linearity layer, and a pooling layer [47]. The input and output of each layer are sets of arrays called feature maps. In the convolution layer, the convolution operation scans the feature maps of previous layer through a set of weights called a filter bank to produce output feature maps using the formula: ${f}_j^n={\sum}_{k=1}^K{f}_k^{n-1}\ast {w}_{kj}^n$, where: ${f}_j^n$ is the output feature map, ${f}_k^{n-1}$ is the input feature map and ${w}_{kj}^n$ is the filter (kernel). All components in a feature map share the same filter bank. Different feature maps in a layer are formed by different filter banks. The output after convolution operation is then passed through a nonlinear activation layer, such as the Rectified Linear Units (ReLU). Compared with traditional tanh or sigmoid functions, the ReLU has more sophisticated non-linearities without suffering from the vanishing gradient problem. The role of the pooling layer is to reduce the dimension of feature maps by merging semantically similar features into one. A typical pooling operation computes the average values, max values or sum values over a region in one feature map. After one, two or more stages of convolution, non-linearity and pooling operations, a fully connected layer receives the output of last stage and passes new output to a soft-max loss function.

### Evaluation of model performance

The trained classification models are evaluated using receiver operating characteristic (ROC) curve and F1 score. Both classification metrics are calculated from true positives (TP), false positives (FP), false negatives (FN) and true negatives (TN). The ROC curve plots the true positive rate (*TPR* = *TP*/(*TP* + *FN*), also called sensitivity), against the false positive rate (*FPR* = *FP*/(*FP* + *TN*)), which equals 1-specificity. The area under the ROC curve is AUC, representing the trade-off between sensitivity and specificity. The maximum value of AUC is 1.0, denoting a perfect prediction, while a random guess gives an AUC value of 0.5. The F1 score balances recall and precision equally and combines them in a single score:

$$ \mathrm{F}1=2\ast \frac{\mathrm{TP}}{2\mathrm{TP}+\mathrm{FP}+\mathrm{FN}} $$

F1 score falls in the interval of [0, 1]. A perfect classifier would reach a score of 1 and a random classifier would reach a score of 0.5.

## Additional file

Additional file 1: **Figure S1.** The one-stage model architecture optimization for activity prediction. **Figure S2.** The two-stages model architecture optimization for activity prediction. **Figure S3.** Comparison of classification performance for different top- and bottom-efficacy cutoffs that used to construct training data set. **Figure. S4.** AUC values and F1 scores comparing the performance of the different “one-hot” encoding modes. **Figure S5.** Higher order features consume more computation time. **Figure S6.** Optimized parameters determination and 5-fold cross validation for the activity prediction using scikit-learn package in python. **Figure S7.** Preference of nucleotide sequences that impact Cpf1 guide RNAs activity. **Figure S8.** The model architecture optimization for specificity prediction. **Figure S9.** Optimized parameters determination and 5-fold cross validation for the specificity prediction using scikit-learn package in python. **Figure S10.** Visualizing and filtering guide RNAs for the TADA1 gene. The optimized 10 guides were chosen based on the high on-target activity and less off-target sites. (DOCX 2931 kb)

## Data Availability

An R software package is available through GitHub at https://github.com/lje00006/DeepCpf1, containing all the source code used to run DeepCpf1.

## Abbreviations

AUC: Area Under the Curve
Boosted RT: Gradient-boosted Regression Tree
Cas: CRISPR-associated Proteins
CNN: Convolution Neural Networks
CRISPR: The Clustered Regularly Interspaced Short Palindromic Repeats
FN: False Negatives
FP: False Positives
KNN: k-nearest neighbor
L1 regression: L1-regularized Linear Regression
L1 L2 regression: L1 L2-regularized Linear Regression
L2 regression: L2-regularized Linear Regression
NN: Neural Network
ReLU: Rectified Linear Units
RF: Random Forest
ROC: Receiver Operating Characteristic
SVM: Support Vector Machine
TN: True Negatives
TP: True Positives
