# Supplementary material for: Prediction of activity and specificity of CRISPR-Cpf1 using convolutional deep learning neural networks
Source: BMC Bioinformatics. 2019 Jun 13;20:332. doi: 10.1186/s12859-019-2939-6 (PMC6567654; doi:10.1186/s12859-019-2939-6)

Supporting material for: Prediction of activity and specificity of CRISPR-Cpf1 using convolutional deep learning neural networks

Jiesi Luo^1,2*^, Wei Chen^2^, Li Xue^3^ and Bin Tang^3*^

1 Key Laboratory for Aging and Regenerative Medicine, Department of Pharmacology, School of Pharmacy, Southwest Medical University, Luzhou, Sichuan, China

2 Center for Bioinformatics and Systems Biology and Department of Radiology, Wake Forest School of Medicine, Winston-Salem, NC 27157, USA

3 School of Public Health, Southwest Medical University, Luzhou, Sichuan, China

4 Basic Medical College of Southwest Medical University, Luzhou, Sichuan, China

* To whom correspondence should be addressed. Email: ljs@swmu.edu.cn

Jiesi Luo

Email address: ljs@swmu.edu.cn

Wei Chen

Email address: [wchen@wakehealth.edu](mailto:wchen@wakehealth.edu)

Li Xue

Email address: lx@swmu.edu.cn

Bin Tang

Email address: bt@swmu.edu.cn

**Supplemental Figure S1. The one-stage model architecture optimization for activity prediction. a Comparison of classification performance for different types of nonlinear activation function and pooling operation. b Comparison of classification performance for different sizes of kernel. c Comparison of classification performance for different numbers of feature maps. d Comparison of classification performance for different numbers of layers.**

**
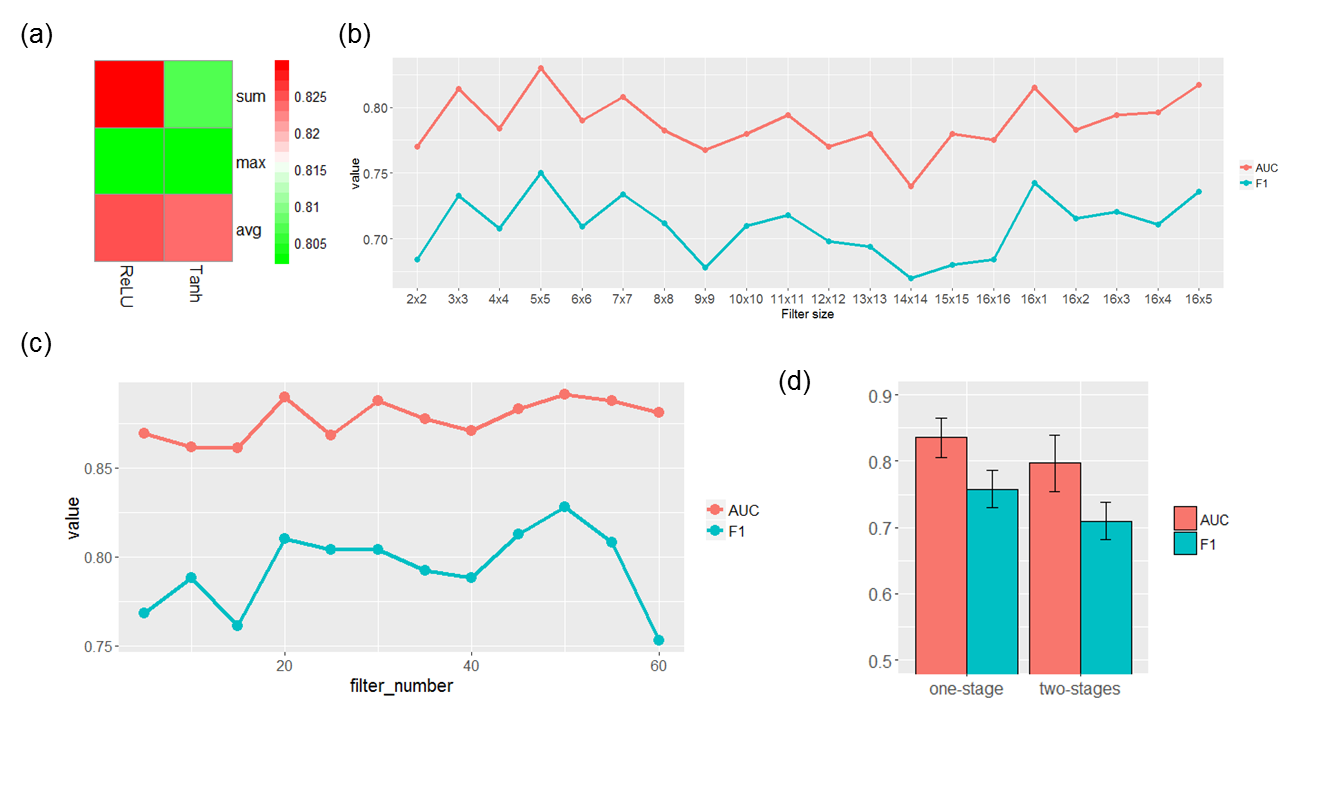
**

**Supplemental Figure S2. The two-stages model architecture optimization for activity prediction. a Comparison of classification performance for different types of nonlinear activation function and pooling operation. b Comparison of classification performance for different sizes of kernel. c Comparison of classification performance for different numbers of feature maps. d Comparison of classification performance for different numbers of fully connected layer neurons. e Full schematic diagram of two-stages network architecture.**

**
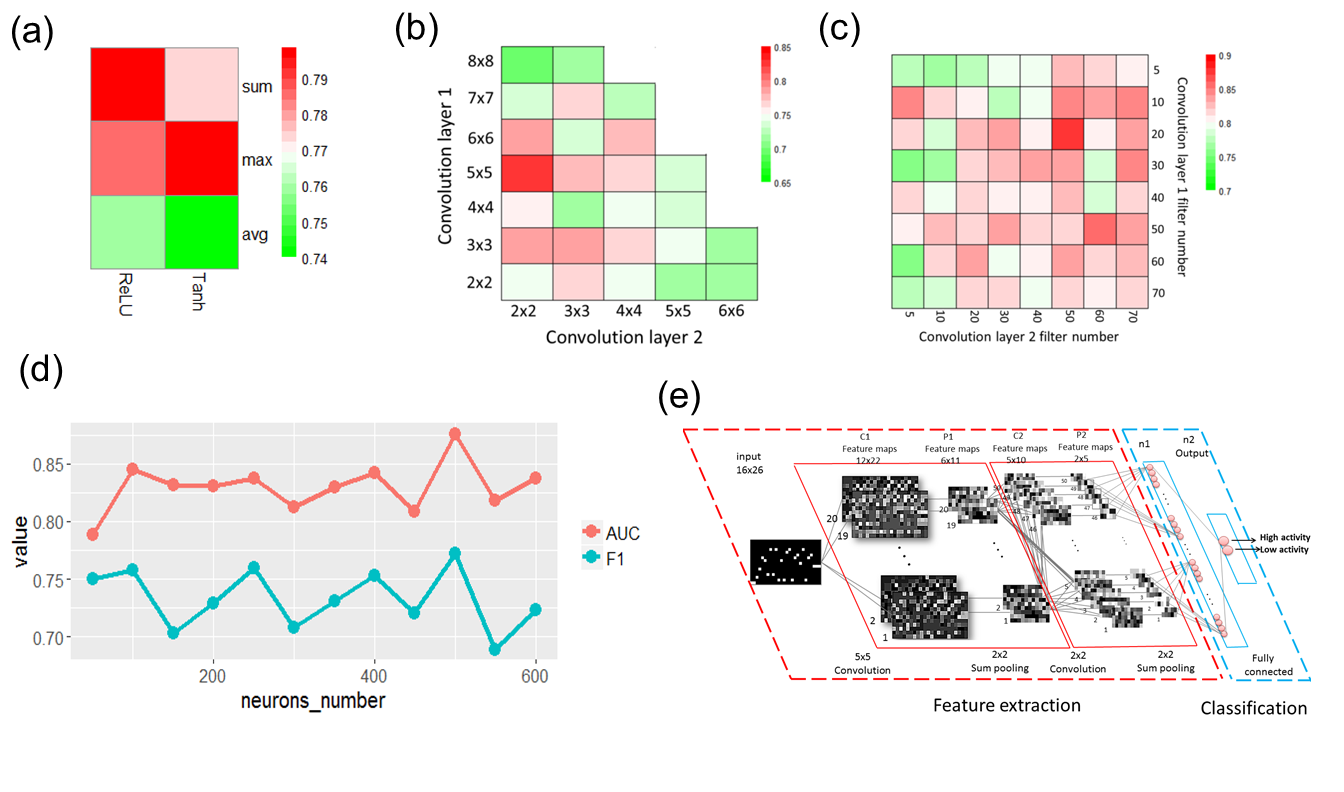
**

**Supplemental Figure S3. Comparison of classification performance for different top- and bottom-efficacy cutoffs that used to construct training data set.**

**
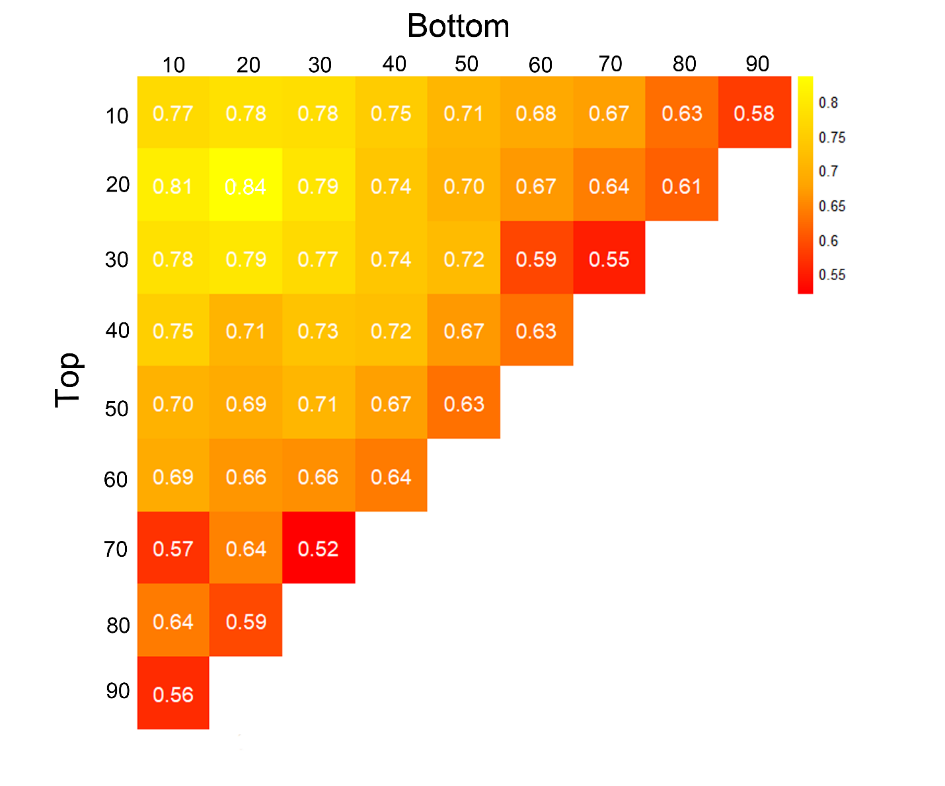
**

**Supplemental Figure S4. AUC values and F1 scores comparing the performance of the different “one-hot” encoding modes.**

**
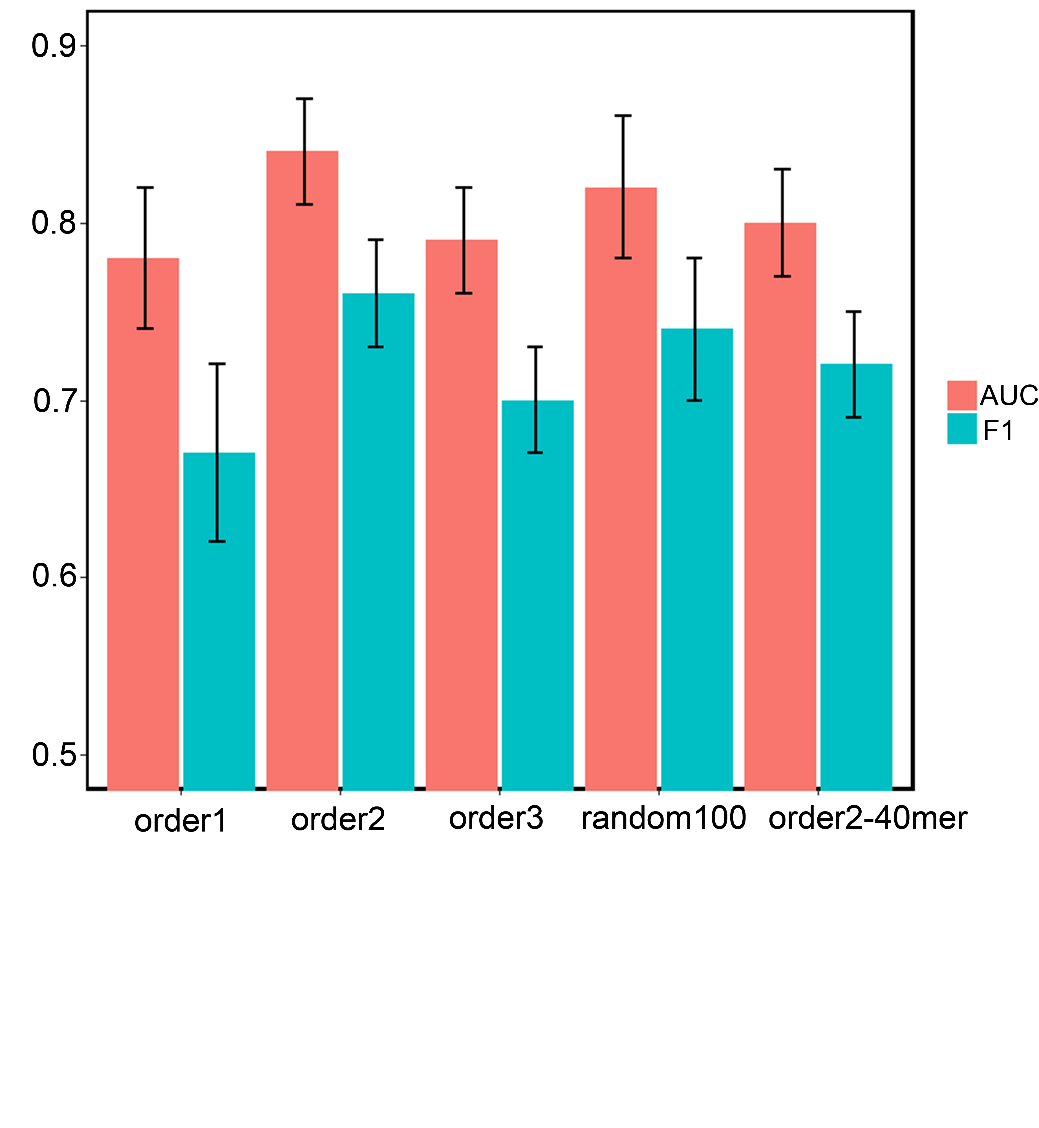
**

**Supplemental Figure S5. Higher order features consume more computation time.**

**
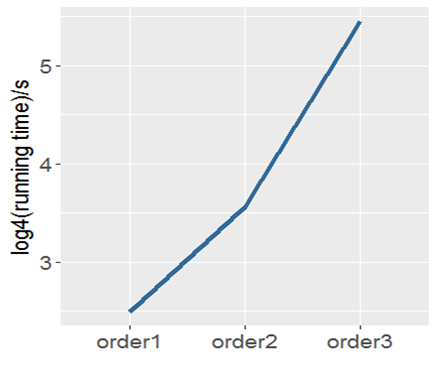
**

**Supplemental Figure S6. Optimized parameters determination and 5-fold cross validation for the activity prediction using scikit-learn package in python. a L1-regularized linear regression (L1 regression), L2-regularized linear regression (L2 regression) and L1L2-regularized linear regression (L1L2 regression). b Support Vector Machine. c Random Forest. d Neural Network and k-nearest neighbor.**

**
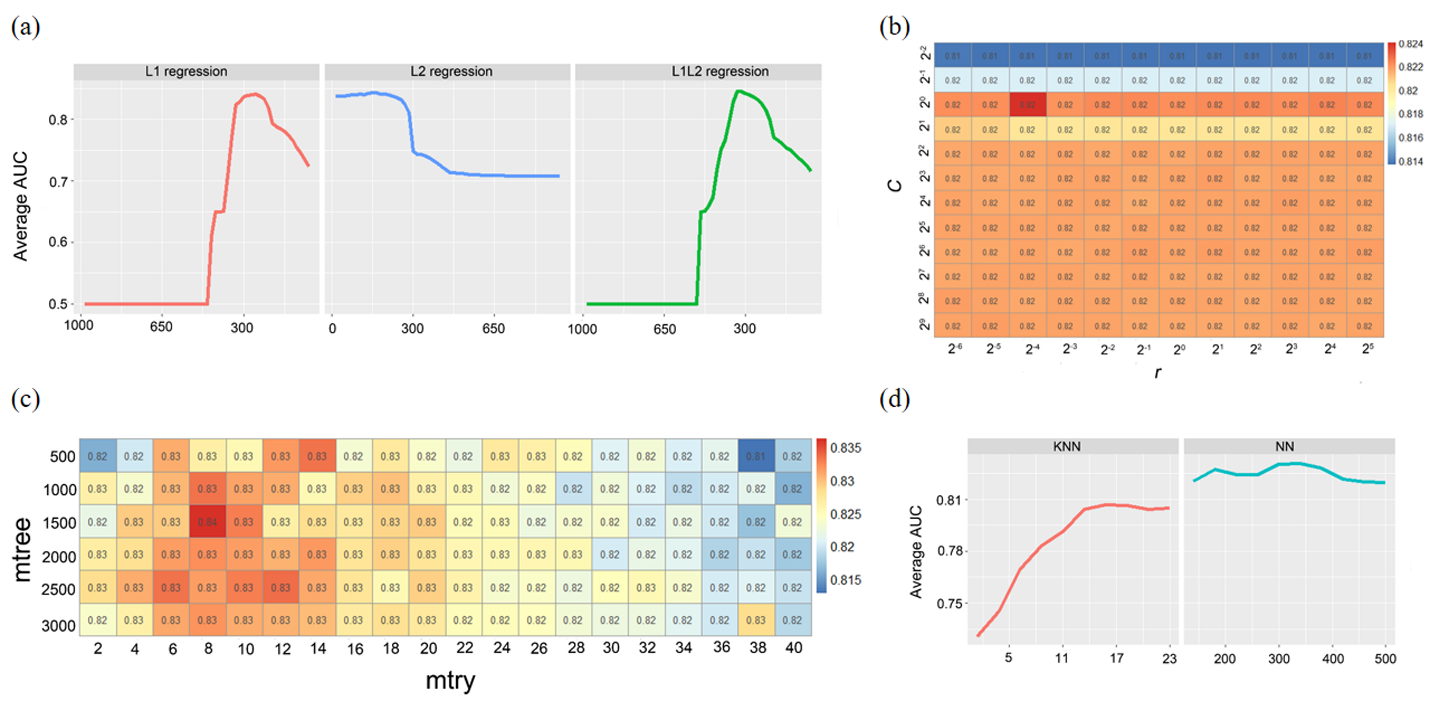
**

**Supplemental Figure S7. Preference of nucleotide sequences that impact Cpf1 guide RNAs activity**


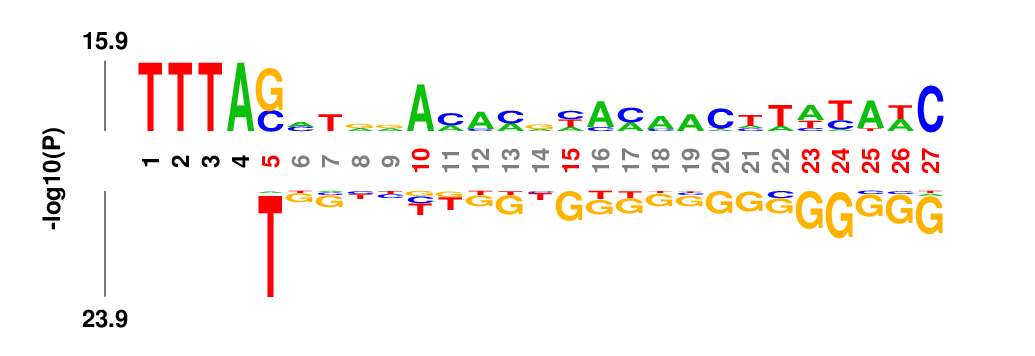


**Supplemental Figure S8. The model architecture optimization for specificity prediction. a Comparison of classification performance for different types of nonlinear activation function and pooling operation. b Comparison of classification performance for different sizes of kernel. c Comparison of classification performance for different numbers of feature maps. d Comparison of classification performance for different row order.**

**
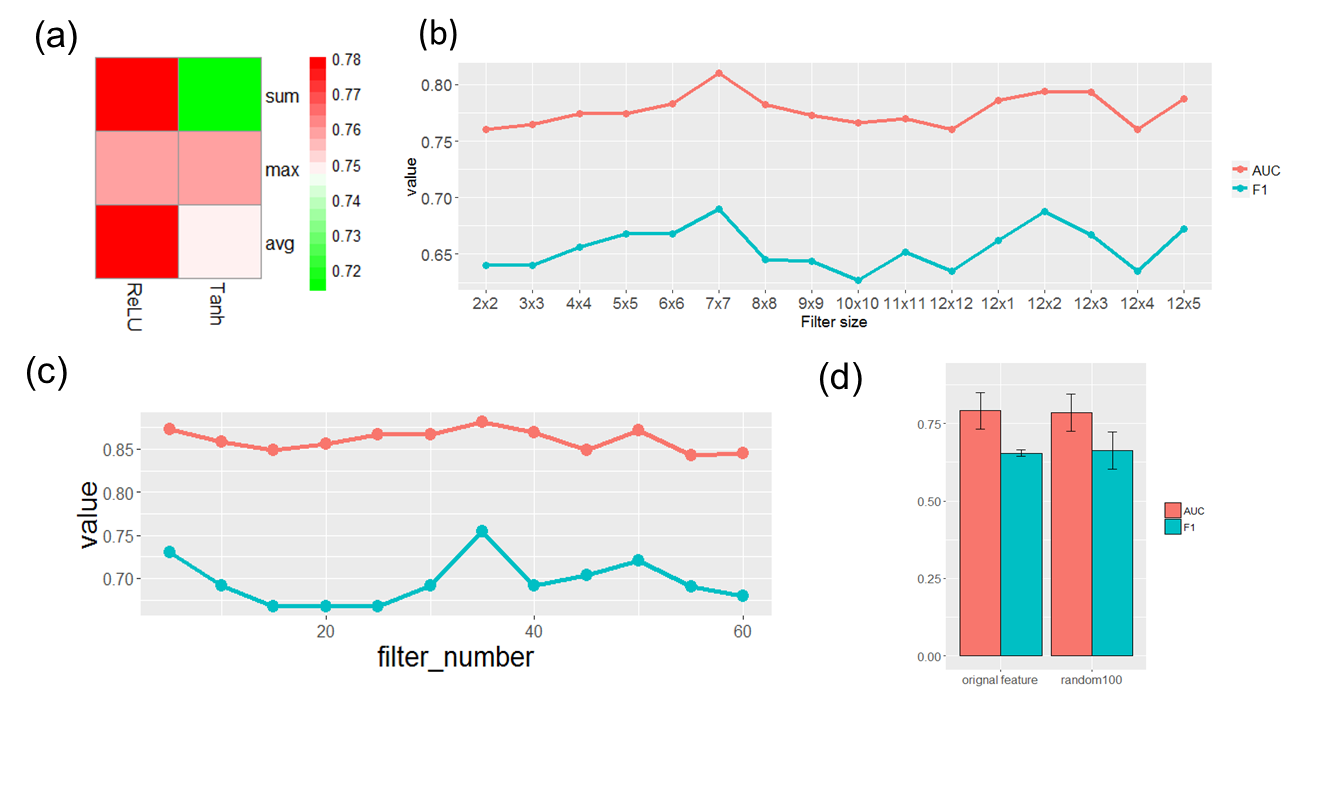
**

**Supplemental Figure S9. Optimized parameters determination and 5-fold cross validation for the specificity prediction using scikit-learn package in python. a L1-regularized linear regression (L1 regression), L2-regularized linear regression (L2 regression) and L1L2-regularized linear regression (L1L2 regression). b Support Vector Machine. c Random Forest. d Neural Network and k-nearest neighbor.**

**
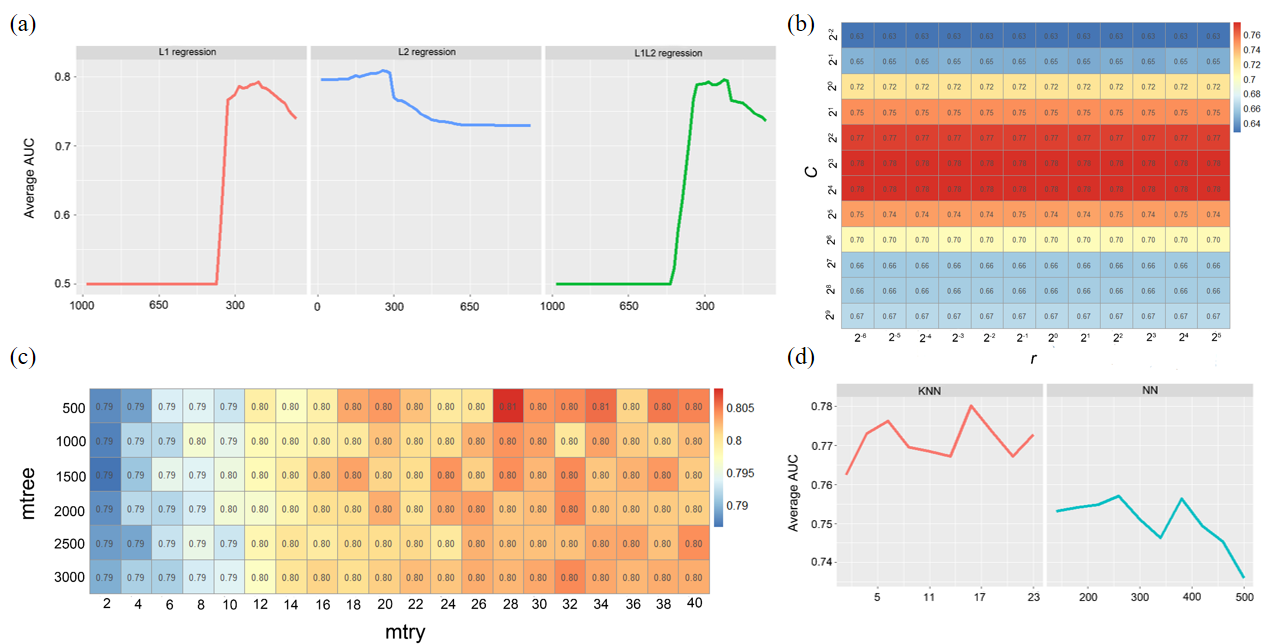
**

**Supplemental Figure S10. Visualizing and filtering guide RNAs for the TADA1 gene. The optimized 10 guides were chosen based on the high on-target activity and less off-target sites**


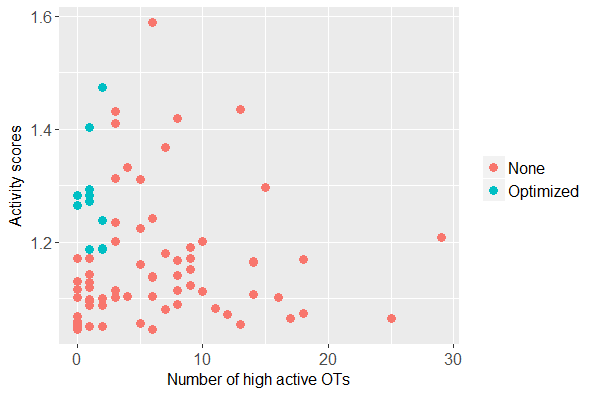

Supplement: Supplementary file 1 — Figure S1. The one-stage model architecture optimization for activity prediction. Figure S2. The two-stages model architecture optimization for activity prediction. Figure S3. Comparison of classification performance for different top- and bottom-efficacy cutoffs that used to construct training data set. Figure. S4. AUC values and F1 scores comparing the performance of the different “one-hot” encoding modes. Figure S5. Higher order features consume more computation time. Figure S6. Optimized parameters determination and 5-fold cross validation for the activity prediction using scikit-learn package in python. Figure S7. Preference of nucleotide sequences that impact Cpf1 guide RNAs activity. Figure S8. The model architecture optimization for specificity prediction. Figure S9. Optimized parameters determination and 5-fold cross validation for the specificity prediction using scikit-learn package in python. Figure S10. Visualizing and filtering guide RNAs for the TADA1 gene. The optimized 10 guides were chosen based on the high on-target activity and less off-target sites. (DOCX 2931 kb) [file 12859_2019_2939_MOESM1_ESM.docx]
